# The Role of Ursodeoxycholic Acid Administration During the COVID-19 Pandemic: A Questionnaire Survey

**DOI:** 10.1155/cjid/4601882

**Published:** 2025-01-13

**Authors:** Cheng Zhou, Ran Jia, Jinqiu Yang, Tong Liu, Xiaoyan Liu, Lin Yang, Wenxia Zhao

**Affiliations:** ^1^Department of Oncology, Wenzhou Hospital of Integrated Traditional Chinese and Western Medicine, Wenzhou 325000, Zhejiang, China; ^2^Key Laboratory of Liver Disease, The First Affiliated Hospital of Henan University of Chinese Medicine, Zhengzhou, Henan, China; ^3^Department of Hepatobiliary Surgery, Xianyang Central Hospital Affiliated to Shaanxi University of Chinese Medicine, Xianyang, Shaanxi, China

**Keywords:** COVID-19, infection control, SARS-CoV-2, surveys and questionnaires, ursodeoxycholic acid

## Abstract

In December 2022, China classified COVID-19 as a category B infectious disease. This ended 2 years of close epidemiological surveillance of COVID-19. The objective of this questionnaire was to assess the infection status in the COVID-19 pandemic since December in Henan Province, China, and the prevalence of infection in people who were taking ursodeoxycholic acid (UDCA) during this period. We distributed questionnaires to patients attending the gastroenterology clinic at the First Affiliated Hospital of Henan University of Chinese Medicine. The questionnaire lasted for 3 weeks and a total of 660 were collected, of which the number of people taking UDCA was 70. This is the first investigation into the rate of infection among those taking UDCA during the time of the COVID-19 pandemic. Our results showed that the overall infection rate among those taking UDCA was 71.43% (*n* = 50), with a 10% (*n* = 7) rate of asymptomatic infections, which was significantly lower than the 85.42% (*n* = 504) and 6.27% (*n* = 37) rates among respondents who did not take. The administration of UDCA showed a trend toward reducing the rate of COVID-19 infection, but the difference was not statistically significant when compared to patients with shorter durations of medication use. While less than 30% of participants remained uninfected during the study period, indicating a potential protective effect, it is important to note that complete prevention of SARS-CoV-2 infection by UDCA was not observed.

## 1. Introduction

The global pandemic of severe acute respiratory syndrome coronavirus 2 (SARS-CoV-2) began in late 2019 and has had a profound impact on global health and socioeconomic systems [1]. As the 2021 vaccination program advances in most countries, the number of confirmed cases begins to show a downward trend. However, new mutant strains, including Alpha and Beta, pose new challenges for outbreak prevention and control efforts. Moving into 2022, the Omicron variant emerged as the dominant epidemic strain, characterized by greater transmissibility but relatively low pathogenicity. Despite increases in vaccination rates, the number of cases remained high globally, especially in those groups with low vaccination rates. By 2023, continued mutation of the virus led to the emergence of new variants such as XBB and BQ.1. The number of cases globally fluctuated but showed an overall downward trend throughout 2023. In the first half of 2024, the number of cases globally continued its downward trajectory. However, a recent WHO report (August 13, 2024) showed a 30% and 26% increase in new cases and deaths, respectively, compared to the previous month (May 27–June 23, 2024). As of July 21, 2024, the cumulative number of confirmed cases reported globally since the beginning of the pandemic has exceeded 775 million and the number of deaths has exceeded 7 million [2]. Epidemic prevention and control efforts continue to require sustained attention and effort.

In late January 2020, COVID-19 was transmitted on a large scale in Wuhan, China. Since then, China has been implementing precise prevention and control of COVID-19. Over the past two years, several minor epidemics have emerged locally in China, but the source of infection was immediately identified and social transmission was quickly interrupted [3]. China commenced mass vaccination against COVID-19 on December 15, 2020 [4]. As of January 20, 2023, 3.488 billion vaccination doses have been administered, with a coverage rate of 92.9% for the first dose and 90.5% for full vaccination among the entire population [2]. Wearing face masks and using ethanol disinfection are effective measures to prevent the spread of COVID-19 [5]. There has been a noticeable increase in the purchase of personal protective equipment since December 2022, leading to a shortage of supply at healthcare facilities. However, the close proximity of social activities and the high population density led to a rapid surge in COVID-19 cases shortly after the restrictions were lifted in China. The number of Internet searches for the epidemic and health consultations peaked on December 21 according to Baidu epidemic index. Hospitalization and readmission rates for Delta and Omicron variants are markedly lower now than in 2020 [6–8]. According to data from the Chinese Center for Disease Control and Prevention [2], the mutant strains in the current pandemic in China are mainly Omicron mutants, with the most common strains being BA.5.2 and BF.7.

Previous studies have demonstrated that angiotensin-converting enzyme 2 (ACE2) serves as the primary pathway through which coronavirus surface spike proteins enter and infect various human cells, including endothelial cells and nerve cells [9]. Theoretically, the invasion of coronaviruses can be halted by modulating the ACE2 receptor. Brevini et al. [10] found that UDCA can close ACE2 pathways by decreasing farnesol receptor X, suggesting that UDCA may have a preventive effect on COVID-19. Although their organoid tests produced the anticipated results, there were no clinical studies to support their findings. Here, we assessed the population prevalence of COVID-19, post-infection manifestations, and hospitalization rate in Henan Province, China, by questionnaire to investigate whether UDCA could be an important agent against SARS-CoV-2.

## 2. Materials and Methods

### 2.1. Study Design and Participants

We conducted an online survey from January 13, 2023, to February 4, 2023. The survey was carried out with Sojump (https://www.wjx.cn). Sojump is a professional online questionnaire survey, evaluation, and voting platform, which provides access to the questionnaire via WeChat. Patients attending the gastroenterology clinic at the First Affiliated Hospital of Henan University of Chinese Medicine were allowed to fill in the questionnaire by scanning the QR code. All participation is voluntary and respondents are assured of confidentiality and anonymity. This was an anonymous questionnaire-based study. Participants scanned a QR code to access the study overview. They could then choose to provide informed consent and continue the questionnaire, with the ability to withdraw at any time during completion. We collected only the gender and date of birth of the participants and did not gather additional personal information such as names. Upon completion of the questionnaire, the software will record the time taken to fill it out and capture the participant's IP address. In addition, the questionnaire content is only accessible to us and not visible to the participants. The study protocol was approved by the Ethics Committee of the First Affiliated Hospital of Henan University of Traditional Chinese Medicine (approval number 2023HL-256). Participants who completed the questionnaire in less than 120 s and those with duplicate IP addresses will be excluded.

### 2.2. Questionnaire Design

**Figure 1:**
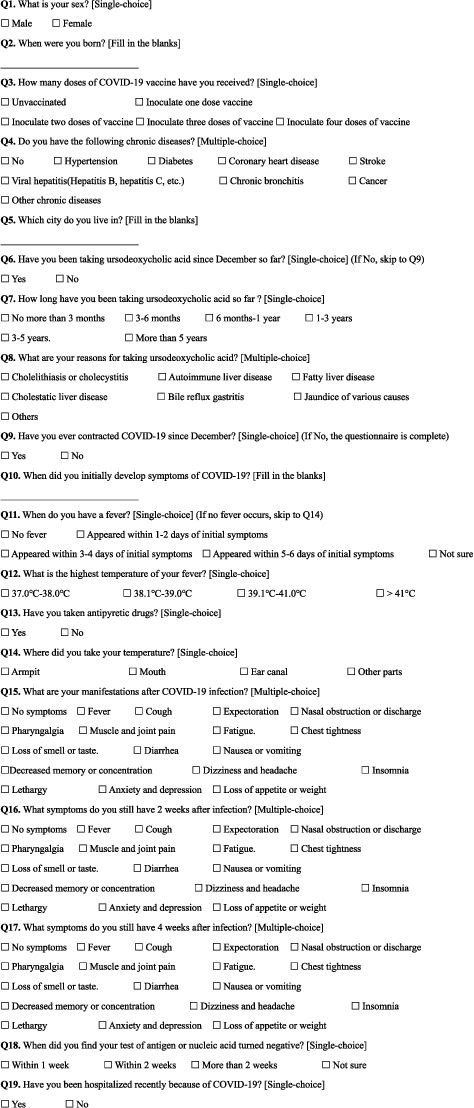
Survey questionnaire.

The construction of the questionnaire was meticulously segmented into several pivotal areas: Section 1 pertains to the acquisition of fundamental demographic data, encompassing inquiries on gender (Q1), birthdate (Q2), vaccination history (Q3), chronic ailment background (Q4), and geographical domicile (Q5). This stratum of inquiry serves to delineate potential correlations between various demographic factors and the incidence of COVID-19 infection, as well as the spectrum of symptomatic manifestations observed in affected individuals.  Section 2 delves into the specifics of UDCA administration, probing for its utilization with or without concurrent intake of UDCA (Q6), the chronicity of its use (Q7), and the underlying motivations prompting its consumption (Q8). This segment is instrumental in uncovering any discernible linkages between UDCA usage and the rates of COVID-19 infection alongside the clinical manifestations thereof. The latter portion of the questionnaire (Q9–Q19) is crafted to chronicle the intricacies of participants' COVID-19 infection trajectories. It encompasses the verification of infection status, a detailed inventory of symptomatology, precise temperature measurements, the chronicity of symptomatic episodes, and the temporal dynamics associated with the transition in viral test outcomes. The questionnaire entries are shown in Figure 1.

### 2.3. Statistical Methods

All statistical analyses were performed using IBM SPSS software (Version 26). Participant characteristics of continuous variables and descriptive statistics were expressed as mean ± standard deviation (SD). Count data were expressed as percentages (%); independent samples *t*-test and ANOVA were used for comparison between groups, and logistic regression analysis was used for multifactor analysis. *p* value of < 0.05 was considered statistically significant.

## 3. Results

### 3.1. Characteristics of the Participants

**Figure 2:**
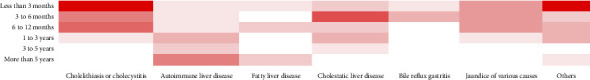
Association between the duration of UDCA administration and the disease. The darker the red, the closer the connection.

In the aggregate dataset of 722 questionnaires, an analysis revealed that 56 responses were completed in a duration of less than 120 s, suggesting potential inattention to the comprehensiveness of the questionnaire content. Additionally, 15 responses were identified as duplicates, originating from 6 distinct IP addresses. Following the application of exclusion criteria to eliminate these responses, a total of 62 questionnaires were deemed invalid. Consequently, the study's analytical cohort was constituted by a total of 660 valid participants, ensuring the robustness and reliability of the subsequent data analysis. In Table 1, we summarize the characteristics of the participants. Of the 660 participants, 70 had been taking UDCA since the outbreak in December, whereas 590 individuals had not. The mean age of UDCA users was 58.49 ± 12.65 years, whereas those who were not taking the medication had a mean age of 40.27 ± 13.19 years. This difference in ages was statistically significant (*p* < 0.01, 95% CI: −21.50, −14.94). In addition, vaccination coverage reached 82.86% (*n* = 58) among participants treated with UDCA, compared with 94.07% (*n* = 555) among those who did not receive UDCA. The difference in vaccination coverage between the two groups was statistically significant (*p* < 0.01, 95% CI: −0.175, −0.049). Among participants who were not taking UDCA, 25.59% (*n* = 151) had at least one chronic disease, while among those taking UDCA, the percentage was 88.57% (*n* = 62), with primary hypertension being the most prevalent chronic disease in both groups (9.83%). In participants administering UDCA, significant disparities were noted in the prevalence of comorbid conditions, including chronic obstructive pulmonary disease (COPD), arterial hypertension, diabetes mellitus, and coronary heart disease, when compared to those not receiving UDCA. Furthermore, an elevated risk of stroke was observed. This observation may be correlated with the advanced age of the patients and is indicative of the demographic profile of this specific population. UDCA is commonly prescribed for the amelioration of symptoms associated with cholestasis, a condition that is frequently concomitant with obesity or immune dysregulation. The pathophysiology of cholestasis in these patients may precipitate a higher prevalence of metabolic syndrome features, which in turn could be linked to the elevated rates of cardiovascular comorbidities [11]. Additionally, cholestasis is often associated with chronic inflammation, a state that may further contribute to the augmented risk of cardiovascular disease [12]. The underlying liver disease itself may contribute to a prothrombotic state and endothelial dysfunction, which are well-established risk factors for stroke. Among 590 participants who did not take UDCA, the observed population prevalence of infection was 85.42% (*n* = 504), with an asymptomatic infection rate of 6.27% (*n* = 37). The cohort of comprising 30 unvaccinated individuals and 5 participants who had received a single vaccine dose exhibited a higher population infection rate of 91.43% (*n* = 32). There appears to be a trend toward increased infection rates among participants with partial vaccination or absent vaccination status. However, statistical significance was not achieved, potentially attributable to the limited sample size of the subgroups (*p* > 0.05, 95% CI: −0.105, 0.093). 70 participants started taking UDCA consistently before the outbreak, taking the drug for reasons including cholelithiasis or cholecystitis, autoimmune liver disease, and cholestatic liver disease. From Figure 2, we can clearly see the association between the duration of UDCA administration and the disease. The infection prevalence was 71.43% (*n* = 50), with a significant difference (*p* < 0.05, 95% CI: 0.478–0.659) versus nonmedicated participants. Asymptomatic cases comprised 10% (*n* = 7), nonsignificantly differing (*p* > 0.05, 95% CI: −0.099 to 0.025). 12 of the participants were unvaccinated and had a population prevalence of 66.67% (*n* = 8), which was also significantly different from the participants who did not take UDCA. Most patients with cholelithiasis or cholecystitis take UDCA for a relatively short time, while patients with autoimmune liver disease take it for a long time. Further analysis revealed that the reason and duration of UDCA administration did not significantly correlate with the infection rate of COVID-19 (*p* > 0.05).

### 3.2. Characteristics of COVID-19 Manifestations

**Figure 3:**
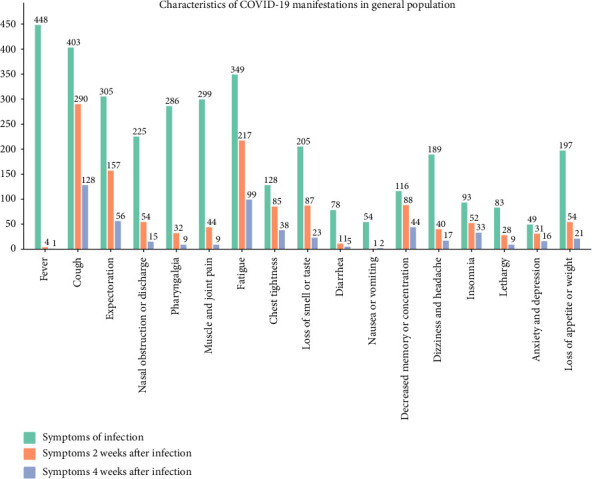
Characteristics of COVID-19 manifestations in general population. Red indicates manifestation characteristics after infection, blue indicates manifestations that are still present 2 weeks after infection, and green indicates manifestations that are still present 4 weeks after infection. Numbers indicate frequency of occurrence.

Among the 504 COVID-19–infected individuals who were not taking UDCA, 63.10% experienced five or more symptoms, with fever (88.89%) being the most prevalent symptom. We found that all patients with a temperature > 41°C took antipyretic drugs, which was also high in patients with high fever (39.1°C-41.0°C) and moderate fever (38.1-39.0°C), 95.49% and 85.23%, respectively. It is notable that the percentage of patients with a maximum body temperature of 37.0°C–38.0°C who used drugs to reduce fever was a remarkable 68.92%. In addition to fever, cough (79.96%), lethargy (56.75%), muscle and joint pain (59.33%), and sore throat (69.25%) were also common clinical manifestations of COVID-19 (Figure 3). In addition to fever, cough (79.96%), lethargy (56.75%), muscle and joint pain (59.33%), and pharyngalgia (69.25%) were also common clinical manifestations of COVID-19 (Figure 3). The persistent symptoms of COVID-19 differed from those that appeared at the beginning of the infection, with only 4 participants still experiencing fever after 2 weeks. After excluding 54 asymptomatic infections or patients whose initial diagnosis was less than 2 weeks, 85 (18.89%) participants recovered from COVID-19 without exhibiting any symptoms after 2 weeks. Cough (79.45%), fatigue (59.45%), expectoration (43.01%), decreased memory or concentration (24.11%), and loss of smell or taste (23.84%) were the most common symptoms after 2 weeks of infection. Excluding 172 asymptomatic infections or patients whose first diagnosis was less than 4 weeks, a total of 150 (45.18%) participants recovered completely after 4 weeks. Cough (70.33%), fatigue (54.40%), expectoration (30.77%), decreased memory or concentration (24.18%), and chest tightness (20.88%) were the most common symptoms 4 weeks after infection. Notably, more than half of the patients still had some symptoms 4 weeks after their initial diagnosis. In addition to respiratory symptoms, continuing neurological symptoms are also common, and the multiorgan damage brought on by the SARS-CoV-2 may significantly affect the patient's quality of life and ability to work.

**Figure 4:**
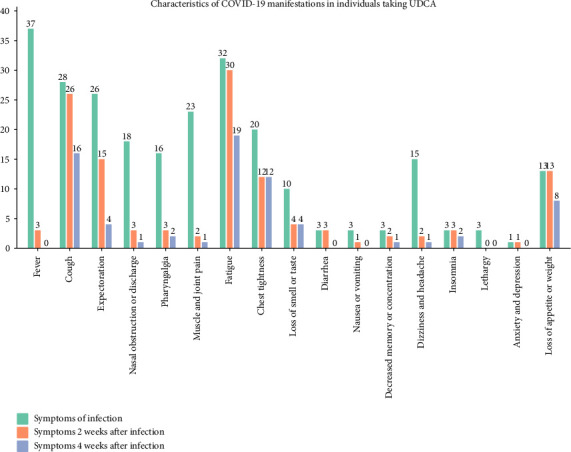
Characteristics of COVID-19 manifestations in individuals taking UDCA. Red indicates manifestation characteristics after infection, blue indicates manifestations that are still present 2 weeks after infection, and green indicates manifestations that are still present 4 weeks after infection. Numbers indicate frequency of occurrence.

Of the 50 COVID-19–infected individuals taking UDCA, 84% had five or more symptoms, which is more severe than in the general population. Fever remains the most frequent symptom in case of infection, with 80% of patients with high fever (39.1°C–41.0°C) and 84% of patients with moderate fever (38.1°C–39.0°C) taking antipyretic drugs. Half of the patients with low fever (37.0°C–38.0°C) also took antipyretic drugs.  Figure 4 shows that individuals taking UDCA initially displayed symptoms that were typical of the general population. However, the systemic symptoms of lethargy, loss of appetite, or weight loss account for a greater proportion of the post-COVID-19 manifestations. After 4 weeks, 63.41% of patients were still experiencing lingering symptoms from COVID-19. Patients taking UDCA exhibit a higher number of symptoms and experience a longer duration of post-COVID-19 manifestations. However, due to the fact that patients taking UDCA are predominantly older individuals with underlying chronic illnesses, post-COVID-19 outcomes may also be influenced by these factors.

## 4. Discussion

In this study, we assessed COVID-19 infection in 660 individuals from Henan, China, and analyzed the differential performance of respondents who were taking UDCA on a continuous basis. There are still no studies revealing the relationship between UDCA and the morbidity of COVID-19, and the association between UDCA and SARS-CoV-2 has only been established in cellular experiments [13] or through computerized virtual models [14, 15]. We analyzed the specific performance of individuals after UDCA administration regarding exposure to SARS-CoV-2 by means of a questionnaire. The continued administration of UDCA did result in a lower rate of COVID-19 prevalence and a greater proportion of asymptomatic infections than in the general population. For post-manifestations of COVID-19 that are not life-threatening but can impair quality of life, patients taking UDCA appear to be more severe and last longer, but this may also be somewhat related to the patient's own chronic disease and age. Our research is essential for the creation of COVID-19 prevention and treatment measures.

After lifting the close monitoring of COVID-19, the prevalence trends were largely similar in all regions of China. In previous studies, the incidence of post-COVID-19 syndrome remained as high as 50.9% 10–14 weeks after infection [16]. In a study of patients hospitalized for COVID-19 [17], more than 30% of patients would be rehospitalized for COVID-19 within 5 months, and more than 10% of patients died as a result. Despite the fact that all available COVID-19 vaccines are very effective against the primary strain and related variations, widespread vaccination was begun in late 2020 [18]. In this study, 92.88% of participants received two or more doses of the vaccine, and 3.07% of patients were admitted to hospitals for COVID-19. Antigen tests turned negative in 46.62% of patients within 1 week. This demonstrates that the majority of patients had a low viral load in the upper respiratory tract and that their bodies are resilient to the virus and healed quickly. In the early outbreaks, the Alpha and Beta strains were more virulent, and the hospitalization and mortality rates were high [19–21]. It is notable that a negative PCR is not the end of patient monitoring. Coronaviruses would deposit in multiple target organs in the body, resulting in numerous post-manifestations of COVID-19. Continuous and long-term monitoring of patients to assess the severity of manifestations after COVID-19, to understand early warning signs, and to intervene in key signs is essential.

SARS-CoV-2, classified as a betacoronavirus with a positive-sense single-stranded RNA genome, exerts its pathogenicity through the interaction of its surface spike glycoprotein with the ACE2 receptor, which is abundantly expressed on the host cell membrane [22]. This precise receptor–ligand engagement facilitates viral entry, subsequent replication, and the initiation of a cascade of immunopathological responses that characterize the clinical spectrum of COVID-19. The current therapeutic landscape for COVID-19 is multifaceted, encompassing both antiviral strategies and host-directed therapies [23]. Antiviral medications constitute a pivotal arm of treatment, with several classes demonstrating efficacy against SARS-CoV-2. These include polymerase inhibitors, protease inhibitors, inhibitors of nucleoside and nucleotide reverse transcriptase, and entry and uncoating inhibitors. Remdesivir, a polymerase inhibitor, has been shown to suppress the activity of the viral RNA-dependent RNA polymerase, thereby exerting its antiviral effect. Clinical trials [24, 25] have indicated that remdesivir significantly reduces hospitalization and mortality rates among COVID-19 patients, leading to its approval by the U.S. Food and Drug Administration (FDA). Azvudine (FNC), a broad-spectrum antiviral agent, has been demonstrated to shorten the time to viral RNA negativity in patients with mild-to-moderate COVID-19 [26, 27]. The National Medical Products Administration of China granted conditional approval for FNC on July 25, 2022, for the treatment of adult patients with mild-to-moderate COVID-19. Host-directed therapies include neutralizing antibody therapy, Janus kinase (JAK) inhibitors, and corticosteroids. The utility of plasma exchange therapy has yielded inconsistent results across large-scale clinical practices globally, leading to inconclusive findings. LY-CoV1404 (bebtelovimab), derived from B cells, is a potent SARS-CoV-2 spike glycoprotein receptor-binding domain (RBD)–specific monoclonal antibody [28]. It has shown significant efficacy in patients with mild-to-moderate COVID-19 and is the only neutralizing monoclonal antibody approved by the FDA for emergency use [29]. The JAK/STAT pathway has been established as a therapeutic target in autoimmune diseases [30, 31]. Baricitinib, a JAK inhibitor, has been shown to significantly reduce the all-cause mortality in hospitalized COVID-19 patients, offering a favorable safety and efficacy profile [32, 33]. Numerous randomized clinical trials [34–36] have substantiated the life-saving potential of corticosteroids, such as dexamethasone and budesonide, in improving the survival rates of severe COVID-19 patients. It is worth noting that blocking the invasion of SARS-CoV-2 into the human body can fundamentally address the spread of COVID-19, and ACE2 may be a promising target. Brevini et al. [10] confirmed this point. However, patients in our study who used oral UDCA for up to 3–5 years were still infected with SARS-CoV-2, and the proportion of infection was not statistically different from that of patients who had been taking the medication for less than 3 months. Only less than 30% of the participants had not been infected with SARS-CoV-2 during this period, indicating that COVID-19 was not completely prevented by administration of UDCA. This could be that standard oral doses cannot entirely block coronavirus binding to the ACE2 receptor, but further research is needed to validate this. In addition to UDCA, a variety of herbs also exhibit high affinity for ACE2 receptors. Lianhua Qingwen Capsule [37] and Qingfei Paidu Decoction [38] are currently recommended formulas in the Chinese guidelines, in which the active ingredients glycyrrhetinic acid and quercetin are involved in the regulation of ACE2 expression [39]. A corpus of randomized controlled trials [40–43] has demonstrated that TCM significantly contributes to the prophylaxis and therapeutics of COVID-19, underscoring its integral role in the contemporary management of this infectious disease.

However, our study has several limitations. Firstly, the study cohort was constrained by a modest sample size, with only 70 participants consistently taking UDCA, which may have introduced a selection bias into our findings. Secondly, the participants in the UDCA group all had pre-existing medical conditions, and there exists a dearth of data pertaining to UDCA usage among healthy individuals. This gap in knowledge limits the generalizability of our results. Furthermore, the reliance on self-reported data through post-infection surveys may have been susceptible to recall bias, potentially compromising the precision of the participants' recollections. The retrospective nature of the data collection could have influenced the accuracy of the responses, as the temporal distance from the infection event and the subjective nature of memory are known to impact the quality of self-reported information. Consequently, there is a clear imperative for future research to adopt more robust methodologies, such as prospective cohort studies or randomized controlled trials, to elucidate the relationship between UDCA usage and COVID-19 outcomes more definitively. Incorporating objective clinical data and minimizing the reliance on participant recall would also serve to bolster the validity of such subsequent inquiries.

## Figures and Tables

**Table 1:** Clinical characteristics of respondents.

Characteristics of respondents	Not taking UDCA	Taking UDCA
Number of patients	590	70
Age, years (mean ± SD)	40.27 ± 13.19	58.49 ± 12.65
Gender, male, *n* (%)	207 (35.08)	38 (54.29)
Vaccination coverage, *n* (%)	555 (94.07)	58 (82.86)
Comorbid disease, at least one, *n* (%)	151 (25.59)	62 (88.57)
Primary hypertension, *n* (%)	58 (9.83)	20 (28.57)
Diabetes, *n* (%)	18 (3.05)	13 (18.57)
Coronary artery disease, *n* (%)	14 (2.37)	6 (8.57)
Chronic obstructive pulmonary disease, *n* (%)	9 (1.53)	5 (7.14)
Viral hepatitis, *n* (%)	35 (5.93)	4 (5.71)
Apoplexy, *n* (%)	2 (0.3)	4 (5.71)
Malignity, *n* (%)	10 (1.7)	4 (5.71)
Others, *n* (%)	57 (9.66)	42 (60)

## Data Availability

The data that support the findings of this study are available from the corresponding authors upon reasonable request.
